# Terlipressin-Induced Skin Necrosis While Managing Hepatorenal Syndrome: A Rare Case Report From North India

**DOI:** 10.7759/cureus.36980

**Published:** 2023-03-31

**Authors:** Sourav Sudan, Sahil Chaudhary, Lakshmi Deepak Bethineedi, Navjot Kaur, Ninia Goyal

**Affiliations:** 1 Internal Medicine, Government Medical College Rajouri, Rajouri, IND; 2 Internal Medicine, Government Medical College Jammu, Jammu, IND; 3 Medicine, Andhra Medical College, Visakhapatnam, IND; 4 Faculty of Medicine and Surgery, Government Medical College Patiala, Patiala, IND; 5 Internal Medicine, Chirayu Medical College and Hospital Bhopal, Bhopal, IND

**Keywords:** skin necrosis, esophageal variceal bleed (evb), esophagial varices, hepatorenal syndrome, terlipressin

## Abstract

Terlipressin is an analogue of vasopressin and is often used in the treatment of bleeding esophageal varices and also in the treatment of hepatorenal syndrome associated with liver cirrhosis. Although terlipressin is a safe drug, but it has been rarely associated with potentially serious adverse effects like ischemic necrosis of skin involving the abdominal skin, extremities, and scrotal skin. We present one such rare case where terlipressin-induced skin necrosis in bilateral lower extremities in a 48-year-old male while we were managing hepatorenal syndrome in the same.

## Introduction

Esophageal variceal rupture and bleeding are major risk factors in cirrhotic patients due to raised portal venous hypertension. Males have a higher risk than females to develop esophageal varices. Esophageal varices are associated with a 50% chance of bleeding in patients and in the following six weeks after an incident of variceal hemorrhage, fatality rates range from 10% to 20% [1]. Esophageal varices bleeding can be prevented by vasoconstrictor medications. Terlipressin is commonly used as a vasoconstrictor.

Terlipressin is an artificial vasopressin analog drug. It causes a reduction in portal venous pressure. It is used as an adjuvant therapy along with banding the esophageal varices. It decreases portal input and blood collection in the splanchnic vasculature by acting on vasopressin 1 (V1) receptors that induce splanchnic vasoconstriction [2]. This lowers portal pressure and enhances glomerular blood flow. Apart from the curative features and benefits, adverse effects are found to be associated with therapy. The adverse effects are due to the vasoconstrictor effects not limited to splanchnic vasculature. The vasoconstriction effect on the heart can lead to myocardial infarction and skin vasculature leads to necrosis of skin tissues [3]. We present one such rare case where terlipressin-induced skin necrosis in bilateral lower extremities in a 48-year-old male while we were managing hepatorenal syndrome (HRS) in the same. 

## Case presentation

A 48-year-old male who was a known case of alcoholic cirrhosis presented to our center with altered mental sensorium for two days associated with a new onset seizure. This was accompanied by a history of constipation for three days. On physical examination, he was having bilateral pedal edema extending up to the knees and there was gross abdominal distension. The vitals at the time of admission included blood pressure = 160/100mmHg, and pulse rate = 80 bpm, temp = 99 ^0 ^F, respirations = 18 per minute. A working diagnosis of hepatic encephalopathy was made and the baseline investigations including the blood workup were ordered. The initial workup revealed decreased serum albumin (2.4mg/dl) and deranged renal function tests (Table 1). Given the abnormal renal function tests in the setting of variceal bleed in a cirrhotic patient, the working diagnosis was changed to HRS.

**Table 1:** Renal and Liver Function Tests.

Parameters	Day one	Day two	Day three
Serum urea	140 mg/dl	135 mg/dl	213 mg/dl
Serum creatinine	4.3 mg/dl	4.1 mg/dl	2.5 mg/dl
Serum sodium	132 mEq/L	132 mEq/L	131 mEq/L
Serum potassium	4.3 mmol/L	3.9 mmol/L	3.5 mmol/L
Serum bilirubin	0.8 mg/dl	2.8 mg/dl	2.7 mg/dl
Alkaline phosphatase	147 U/L	133 U/L	145 U/L
Aspartate transaminase	92 U/L	134 U/L	153 U/L
Alanine transaminase	120 U/L	74 U/L	67 U/L

**Figure 1:**
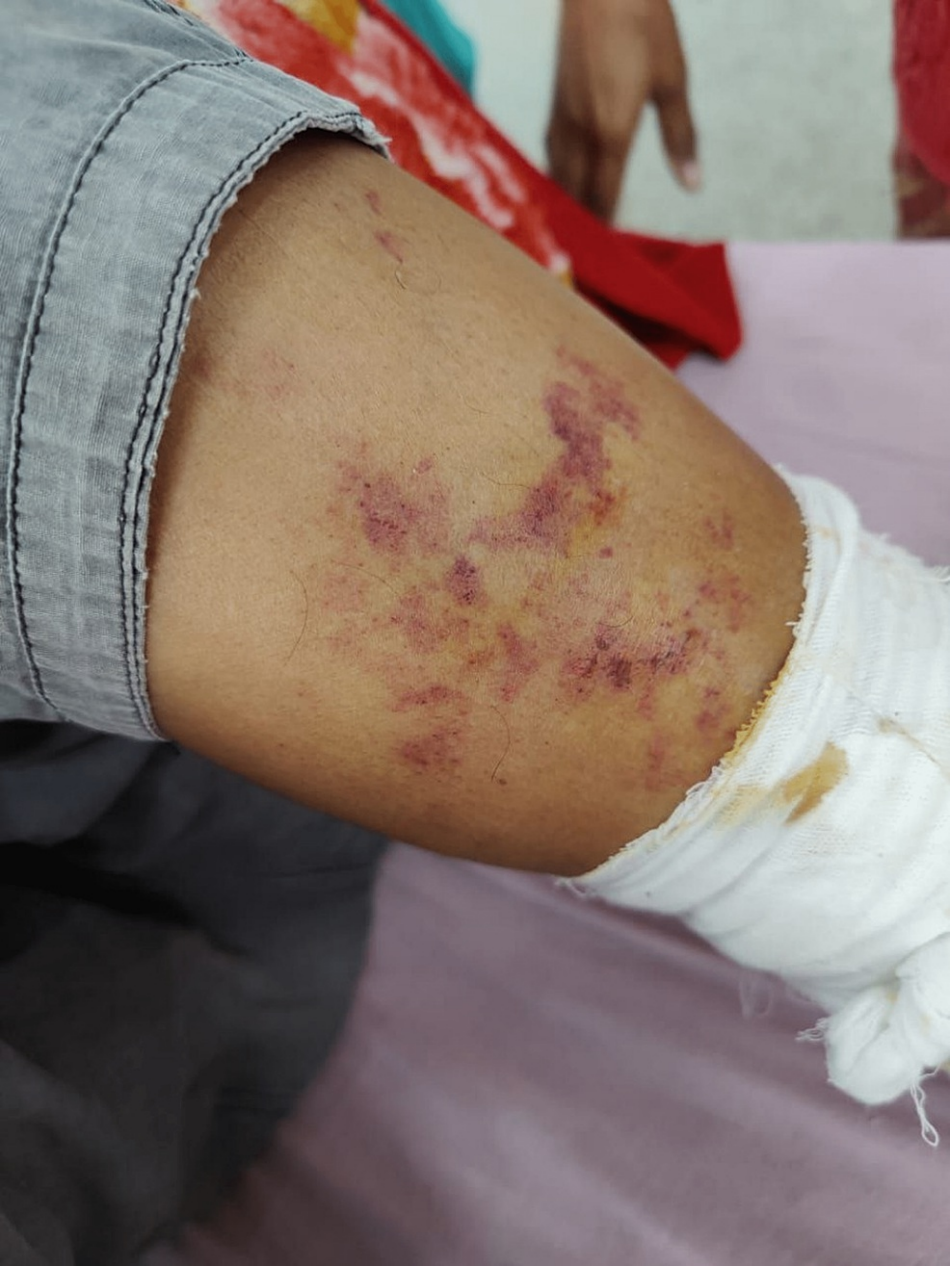
Beginning of development of skin lesions.

Given this diagnosis, we started the patient on human albumin (1g/kg ) and terlipressin (2g every four hours). Within 48 hours of administration of terlipressin, the patient developed reddish-black discoloration of bilateral lower limbs (Figure 1).

**Figure 2:**
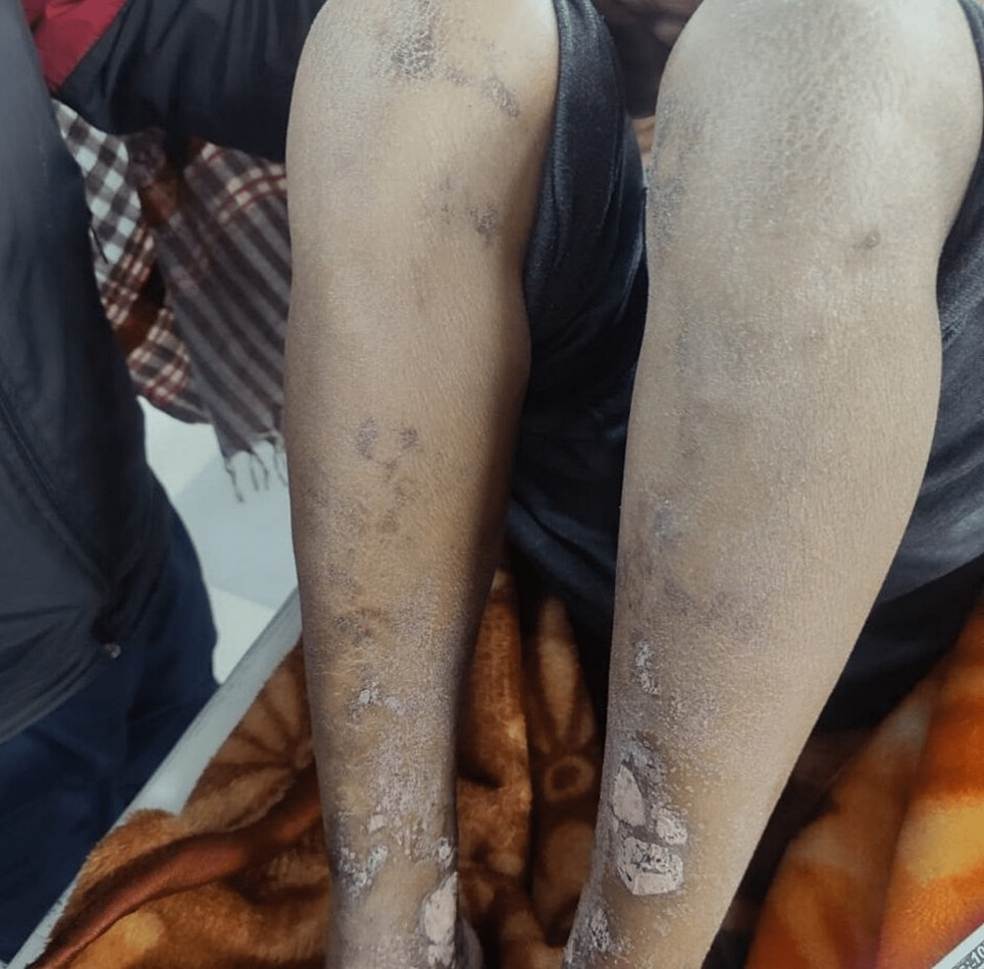
Complete healing of skin lesions after stopping terlipressin.

Bilateral lower limb arterial and venous doppler showed normal patency and normal flow. The vasculitis workup including anti nuclear antibody (ANA), perinuclear anti-neutrophil cytoplasmic antibodies (pANCA), cytoplasmic antineutrophil cytoplasmic autoantibody (cANCA) was done which came out negative. Platelet count was within normal limits. Prothrombin time (PT) and partial thromboplastin time (PTT) were also within normal limits. A skin biopsy was done which showed non-specific changes. A provisional diagnosis of terlipressin-induced cutaneous skin necrosis was made and terlipressin was withheld. We discharged the patient after ensuring normal renal function test and optimal cognitive functioning. The patient was followed up after a week and there was a complete resolution of the rash and healed lesions were seen (Figure 2).

## Discussion

Terlipressin is a well-established drug used in the treatment of bleeding esophageal varices and hepatorenal syndrome [4] associated with liver cirrhosis. Despite its effectiveness, it has been reported to have some adverse effects, including ischemic necrosis, predominantly skin affecting skin over the abdomen, torso, scrotal area, upper and lower extremities [5]. In this case, we present a rare instance where a 48-year-old male patient developed skin necrosis in bilateral lower extremities after administration of terlipressin while managing hepatorenal syndrome.

The mechanism by which terlipressin causes ischemic necrosis of the skin is through its vasoconstrictive properties. Terlipressin acts on the V1 receptors that cause splanchnic vasoconstriction, thereby reducing portal pressure and enhancing glomerular blood flow [6]. However, its vasoconstrictive effect is not limited to the splanchnic vasculature and can extend to the skin vasculature, leading to skin necrosis [3]. The skin necrosis observed in our patient was a result of the patient being administered terlipressin. This was determined by first eliminating other potential causes of skin necrosis, such as vasculitis [7]. To confirm this diagnosis, a skin biopsy was conducted, which revealed non-specific changes, indicative of terlipressin-induced skin necrosis. In addition, the results of the workup for vasculitis, which included tests for ANA, pANCA, and cANCA, were negative. These negative results further reinforced our conclusion that the skin necrosis was indeed caused by terlipressin.

The discontinuation of terlipressin is crucial in the treatment of skin necrosis [8] as it can prevent further progression of the condition and promote healing. Withholding the drug allows the skin to recover and promotes the restoration of blood flow to the affected area. In addition to discontinuing the drug, supportive care is also important in the management of skin necrosis. This may include wound care, such as the application of antiseptic ointments, the use of dressings to protect the affected area, and keeping the area clean and dry. This type of care helps to prevent infection and promote healing. In the case described, the patient was successfully managed by discontinuing the use of terlipressin and providing supportive care. The patient showed complete resolution of the rash and healed lesions within a week of follow-up, demonstrating the effectiveness of this approach. Discontinuation of terlipressin has shown complete resolution of necrosis of skin in various previous case reports [9-12].

## Conclusions

In conclusion, terlipressin-induced skin necrosis is a rare but potentially serious adverse effect that can occur with the use of this drug in the treatment of bleeding esophageal varices and hepatorenal syndrome. It is crucial for healthcare providers to be aware of this adverse effect and to consider alternative treatments in cases where skin necrosis is suspected. In cases where terlipressin is necessary, close monitoring for any signs of skin necrosis should be performed, and the drug should be discontinued immediately if skin necrosis is suspected. The management of skin necrosis involves withholding terlipressin and providing supportive care to prevent further progression and promote healing. Although terlipressin is an effective treatment for bleeding esophageal varices and hepatorenal syndrome, healthcare providers should be vigilant in monitoring for potential adverse effects, including skin necrosis, and consider alternative treatments where necessary.
